# Evaluation of Depigmenting Activity by 8-Hydroxydaidzein in Mouse B16 Melanoma Cells and Human Volunteers

**DOI:** 10.3390/ijms10104257

**Published:** 2009-11-20

**Authors:** Sorgan Shou-Ku Tai, Ching-Gong Lin, Mon-Han Wu, Te-Sheng Chang

**Affiliations:** 1 Department of Biotechnology, National Formosa University/ 64, Wun-Hua Road, Huwei, Yunlin, Taiwan; E-Mail: sorgan@nfu.edu.tw (S.S.-H.T.); 2 Department of Cosmetics Science, Chia Nan University of Pharmacy and Science/ 60 sec.1 Erh-Jen Road, Jen-Te, Tainan, Taiwan; E-Mail: ncglin@mail.chna.edu.tw (C.-G.L.); 3 Sustineo Biotechnology Co., Ltd./ 24 Tai-Chi Road, Kaohsiung, Taiwan; E-Mail: mon-han.wu@sustineo-bio.com (M.-H.W.); 4 Department of Biological Science and Technology, National University of Tainan/ 33 sec. 2 Shu-Lin St., Tainan, Taiwan

**Keywords:** 8-hydroxydaidzein, suicide substrate, skin whitening, tyrosinase inhibitor

## Abstract

In our previous study, 8-hydroxydaidzein (8-OHDe) was demonstrated to be a potent and unique suicide substrate of mushroom tyrosinase. In this study, the compound was evaluated for *in vitro* cellular tyrosinase and melanogenesis inhibitory activities in mouse B16 melanoma cells and for *in vivo* skin-whitening activity in human volunteers. Tyrosinase activity and melanogenesis in the cell culture incubated with 10 μM of 8-OHDe were decreased to 20.1% and 51.8% of control, respectively, while no obvious cytotoxicity was observed in this concentration. In contrast, a standard tyrosinase inhibitor, kojic acid, showed 69.9% and 71.3% of control in cellular tyrosinase and melanogenesis activity, respectively, at a concentration as high as 100 μM. Hence, 8-OHDe exhibited more than an inhibitory effects on melanin production in B16 cells 10-fold stronger than kojic acid. In addition, when a cream containing 4% 8-OHDe was applied to human skin in an *in vivo* study, significant increases in the dL*-values were observed after three weeks. Moreover, the increase in the dL*-values after 8-week treatment with 4% 8-OHDe (from −0.57 to 1.94) is stronger than those of 2% 8-OHDe treatment (from 0.26 to 0.94) and 2% ascorbic acid-2-glucoside treatment (from 0.07 to 1.54). From the results of the study, it was concluded that 8-OHDe, the potent suicide substrate of mushroom tyrosinase, has depigmenting activities in both mouse melanoma cells and in human volunteers. Thus, the compound has significant potential for use in cosmetics as a skin-whitening ingredient.

## 1. Introduction

Mammalian skin color is mainly determined by the content of the pigment melanin, which is produced in unique organelles of the neurocrest-derived melanocyte in skin [1]. Melanin plays an important role in protecting human skin from the harmful effects of UV radiation from the sun. In addition, melanin also determines our phenotypic appearance. Melanin biosynthesis, melanogenesis, is initiated with the first step of tyrosine oxidation to dopaquinone catalyzed by tyrosinase. This first step is the rate-limiting step in melanin synthesis because the remainder of the reaction sequence can proceed spontaneously at a physiological pH value.

Although melanin has mainly a photoprotective function in human skin, the accumulation of an abnormal amount of melanin in different specific parts of the skin resulting in more pigmented patches may become an esthetic problem. The phenomenon has encouraged researchers to seek new potent tyrosinase inhibitors, which inhibit tyrosinase activity and then reduce melanogenesis, for use as the main ingredient in skin-whitening cosmetics. Until now, many tyrosinase inhibitors from natural and synthetic sources have been discovered and reviewed [2]. In addition, some of them have been intensively studied in human clinical trials, and showed whitening effect in skin of the tested volunteers [3–6].

In contrast to the huge number of reversible tyrosinase inhibitors that has been identified, irreversible inhibitors of tyrosinase have rarely been found until now [2]. These irreversible inhibitors can form an irreversibly covalent bond with tyrosinase and then inactivate it. Among the irreversible inhibitors, suicide substrates belong to a special class. The mechanism of the tyrosinase suicide substrate has been extensively studied by Land *et al.*, who demonstrated that suicide inactivation is the result of catecholic substrates being processed by the cresolase route [7]. Because the inhibitory mechanism of the suicide substrates is completely different from those of reversible inhibitors and displays irreversible and partition ratio-dependent inhibition, it has been proposed that the suicide substrates have superior performance in skin-whitening activity than those of usual tyrosinase inhibitors [2]. However, potent suicide substrates have rarely been discovered, and no suicide substrate has been confirmed in depigmenting activity either in cell cultures or in human skin.

**Figure 1.**
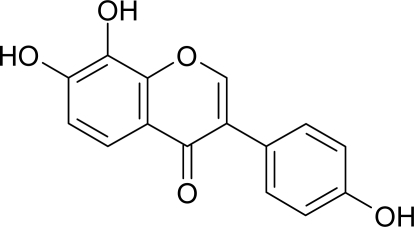
Chemical structure of 8-OHDe.

In our continuing search for new tyrosinase inhibitors, some have been found [8–11]. Among them, 8-hydroxydaidzein (8-OHDe, Figure 1) was recently identified as a potent and unique suicide substrate of mushroom tyrosinase with a low partition ratio, a low Michaelis constant, and a high maximal inactivation rate constant [8–9]. The compound is a biotransformed metabolite of the soy isoflavone daidzein by fungi *Aspergillus saitoi* or *A. oryzae* [12]. In the reviewed literatures, 8-OHDe has been identified as one of the most potent suicide substrates of mushroom tyrosinase until now and has significant potential in application as a skin-whitening agent. Hence, evaluating the depigmenting activity of the compound becomes an interesting issue. In this study, 8-OHDe was evaluated for *in vitro* cellular tyrosinase and melanogenesis inhibitory activities in mouse B16 melanoma cells and for *in vivo* skin-whitening activity in human volunteers, and the depigmenting activities of the compound in both assay systems were confirmed.

## 2. Results and Discussion

### 2.1. In Vitro Evaluation of Depigmenting Activity of 8-OHDe in Mouse B16 Melanoma Cells

**Figure 2.**
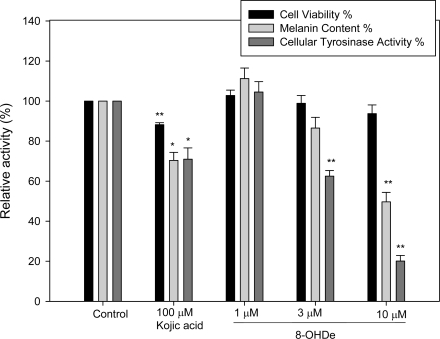
Effects of 8-OHDe on cell viability, melanin content, and cellular tyrosinase activity of mouse B16 melanoma cells. The cells were cultured in 6-well plates and incubated with tested agents for 3 d. Cell viability, cellular tyrosinase activity, and melanin content were assayed as described in the Experimental section. Bars represent the means ± S.D. of three independent experiments. Significant differences were determined by Student’s *t*-test; * *p* < 0.001; ** *p* < 0.0001 compared to control.

Before the *in vivo* skin-whitening assay was conducted, mouse B16 melanoma cells were utilized as a cellular assay system to evaluate the depigmenting activity of 8-OHDe in the cell cultures. We used kojic acid as positive control in the cellular study due to the potent and known inhibitory effects on tyrosinase activity. First, 8-OHDe was applied to these cells at concentrations of 1–10 μM for 48 h, and cell viability was assessed by the MTT method. As shown in Figure 2, at the concentration of 10 μM of 8-OHDe, the cell viability retained 93.7%, which had no statistically significant difference compared to control. Hence, it was concluded that 8-OHDe did not exert cytotoxicity against B16 cells below 10 μM. To investigate whether 8-OHDe exerts depigmenting activity on B16 cells, the change in the melanin contents of the cells treated with 8-OHDe was evaluated. The result showed that melanin contents in the cells with 8-OHDe treatment were significantly reduced in a dose-dependent manner (Figure 2), and the 50% inhibitory concentration was 10.54 μM by the compound. However, kojic acid inhibited melanogenesis of mouse B16 melanoma cells only to 71.7% even at as high a concentration as 100 μM. In addition, kojic acid also had slight cytotoxicity to the mouse B16 melanoma cells. Therefore, 8-OHDe, which exhibited more than a 10-fold stronger inhibitory effects on melanin production in B16 cells than the standard tyrosinase inhibitor, kojic acid, in view of the IC_50_ values, is regarded as a promising skin-whitening agent. This result encouraged us to explore the depigmenting activity of 8-OHDe on human skin in an *in vivo* study.

In addition, the effective concentration of 8-OHDe on inhibition of melanogenesis in B16 melanoma cells was not cytotoxic to the cells. This result suggested that the inhibitory effect of 8-OHDe on melanin biosynthesis is not due to its cytotoxicity. To investigate the inhibitory mechanism by 8-OHDe in reducing melanin contents in B16 melanoma cells, we examined the effect of the compound on activity of the key melanogenic enzyme, tyrosinase. We found that the cellular tyrosinase activity in B16 cells was strongly inhibited by 8-OHDe, and only 20.1% residual tyrosinase activity was retained in the treatment of 10 μM (Figure 2). 8-OHDe also decreased the cellular tyrosinase activity in a dose-dependent manner, and the 50% inhibitory concentration of the compound was 6.17 μM. From the results above, it was concluded that 8-OHDe inhibited melanogenesis in B16 cells due to its effects on reduction of tyrosinase activity. Because 8-OHDe has been proven to be a potent suicide substrate of mushroom tyrosinase, we suggested that the compound reduced cellular tyrosinase activity in B16 cells due to its suicidal property against tyrosinase. However, some major differences exist between the mushroom tyrosinase and the mammalian one. For examples, the mushroom tyrosinase is a cytosol enzyme while the mammalian tyrosinase is membrane bonded. In addition, mushroom tyrosinase is a tetramer in contrast to the monomer type of the mammalian enzyme, which is highly glycosylated during its complex maturation process. Hence, application of the result from the study using mushroom tyrosinase to explain the result from the mammalian study takes some risk. However, to date, no kinetically inhibitory study of tyrosinase activity has been performed with mammalian tyrosinase due to the lack of a purified enzyme. Although some studies used a crude extract of melanocytes as the enzyme source to study simple inhibitory effects of tyrosinase inhibitors, it is difficult to study the kinetic and mechanistic characterizations of the inhibitors to the specific enzyme in the very complex and crude system. In addition, other possible reasons for the reduction of the cellular tyrosinase activity by 8-OHDe in B16 cells, such as inhibition of either tyrosinase gene expression or tyrosinase protein maturation, cannot be ruled out based on the results of the present work. Hence, more detailed experiments for determining the molecular mechanism of 8-OHDe on reduction of cellular tyrosinase activity in B16 cells are needed in the future.

### 2.2. In Vivo Evaluation of Skin-Whitening Activity of 8-OHDe in Human Volunteers

To evaluate the skin-whitening activity of 8-OHDe in advance, we applied the compound on human skin in an *in vivo* study. We used a Chromameter CR-200 photometer to evaluate the skin-whitening activity of 8-OHDe *in vivo*. The skin-whitening index, Luminance (L*)-value of the CIELab space, measured by the machine, has been used by dermatologists as an indicator of skin color for a long time [13]. The L*-value, ranging from total black (L* = 0) to total white (L* = 100), gives the relative brightness of the tested skin. Hence, the increased L*-value of a tested volunteer after drug treatment means her skin color is becoming whiter. In addition, kojic acid, which was used as positive control in the cellular assay system in the present study, has been reported to exert carcinogenic effects on rats and mice [14], and been limited in use on human skin both in European and Taiwan. Hence, in the *in vivo* assay on human volunteers in the present study, we did not use kojic acid as positive control for safety concerns. In contrast to kojic acid, ascorbic acid and its derivatives including magnesium l-ascorbyl-2-phosphate (VC-PMG) and ascorbic acid-2-glucoside (AA2G) are very safe compounds for human use and also the most famous skin-whitening ingredients used in commercial cosmetics [15–18]. Among them, AA2G is the newest ascorbic acid derivative and was demonstrated to be superior to its precursor ascorbic acid in both stability and drug release [16]. For the reasons, we used AA2G as the positive standard in the *in vivo* skin-whitening assay of the present study. All subjects passed the occlusive single patch test for irritation upon exposure to cream containing 4% of 8-OHDe and 2% AA2G and thus were included in the study. The initial skin-whitening index L*-value was taken from the cheek of each subject before application of the tested substances. The tested substances were then applied twice daily on the outer surface of the skin area of each volunteer. Application continued for eight weeks, and the skin-whitening index was measured every week. For data representations every week, the difference between the mean L*-value of drug-treated volunteers and that of placebo-treated volunteers was calculated as the δL*-value of the drug treatment.

**Figure 3.**
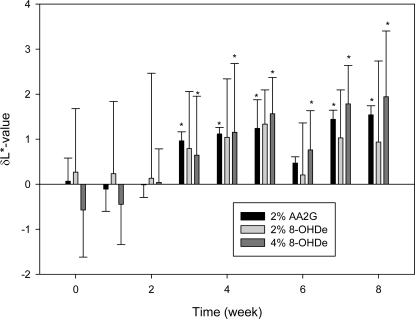
Time course versus δL*-values of different drug treatments. The δL*-value of a drug treatment was calculated from the difference between the mean L*-value of drug-treated volunteers and that of placebo-treated volunteers every week. Bars represent means ± S.D. of data from the total number of volunteers in the tested concentration. Significant differences were determined by Student’s *t*-test; * *p* < 0.01 compared to that at time zero.

Figure 3 shows the representative plots of the time course versus δL*-values of the different drug treatments. From the result of the positive control (2% AA2G treatment), it could be seen that the δL*-values increased with the treatment duration, *i.e.,* from 0.07 at starting to 1.54 at week 8. During the same period, the δL*-values in both the 2% and 4% 8-OHDe treatments also increased with the treatment duration, *i.e.,* from 0.26 at beginning to 0.94 at week 8 for 2% 8-OHDe treatment and −0.57 at beginning to 1.94 at week 8 for 4% 8-OHDe treatment. Among them, the increase in the δL*-values after 8-week treatment with 4% 8-OHDe (from −0.57 to 1.94) is stronger than those of 2% 8-OHDe treatment (from 0.26 to 0.94) and 2% AA2G treatment (from 0.07 to 1.54). When the paired *t*-test was applied to the whitening data at various weeks compared with that at starting, significant differences were found after 3-weeks treatment in both 4% 8-OHDe treatment and 2% AA2G treatment. The result of the 2% AA2G treatment is comparable with the previous one in which the skin-whitening index L*-values of the treated volunteers increased significantly (from −1.14 to 0.36) after 12 weeks of treatment with 10% AA2G [18]. However, the δL*-values in 2% 8-OHDe treatment during 8 weeks contained no significant difference compared with that at beginning. We suggested that the lower statistic data was caused by the lower concentration in use, because significant differences were found in the δL*-values of the 4% 8-OHDe treatment after three weeks. From the results above, it could be concluded that the 4% 8-OHDe indeed caused skin whitening in the 15 volunteers after eight weeks of treatment and performed superior depigmenting activity than those of the 2% of 8-OHDe and AA2G.

## 3. Experimental Section

### 3.1. 8-OHDe Purification and the Cream Formulation

8-OHDe was purified from soy germ koji fermented with *A. oryzae* BCRC 32288, which was obtained from the Bioresources Collection and Research Center (BCRC, Food Industry Research and Development Institute, Hsinchu, Taiwan, ROC). The procedure was as described in our previous work [8]. The purified compound was dissolved in dimethyl sulfoxide (DMSO) and added into cell cultures. For *in vivo* skin-whitening assays, the powdered compound was added into an acidic basal cream to the tested concentration based on its instability in alkaline solution [19]. The tested cream was prepared freshly every month during the *in vivo* assay. The ingredients of the basal cream was composed of glyceryl stearate (3%), liquid paraffin (5%), isopropyl myristate (5%), jojoba oil (2%), glycerin (5%), amphisol (0.5%), germaben II (0.7%), and deionized water to 100%.

### 3.2. Cell Cultures

Mouse B16 melanoma cells (4A5) were obtained from BCRC. The cells were cultured in Dulbecco’s modified Eagle’s medium (DMEM) supplemented with 10% (v/v) fetal bovine serum, at 37 °C in a humidified, CO_2_-controlled (5%) incubator. The cells were seeded at an appropriate cell density in a 6-well plate. After 24 h of incubation, cells were treated with various concentrations of the sample for 48 h in the experiments of cell viability and for 72 h in melanin content and cellular tyrosinase activity assays.

### 3.3. Measurements of Cell Viability

MTT assay was performed to examine the viability of cells. At the end of the drug treatment, the cells were incubated with MTT solution for 4 h, and the optical density of each well was read at 540 nm by a spectrophotometer.

### 3.4. Determination of Melanin Content

The B16 cells were washed with phosphate buffer saline (PBS) at the end of the drug treatment and dissolved in 1 N NaOH containing 10% DMSO for 1 h at 60 °C. The absorbance at 490 nm was measured, and melanin content was measured using the authentic standard of synthetic melanin (Sigma-Aldrich, St Louis, MO, USA).

### 3.5. Measurements of Tyrosinase Activity

To measure the tyrosinase activity of the cells, the B16 cells were lysed by incubation at 4 °C for 30 min in lysis buffer (20 mM sodium phosphate, pH 6.8, 1% Triton X-100, 1 mM PMSF, 1 mM EDTA) containing protease inhibitors cocktail (Abcam, Cambridge, UK). The lysates were centrifuged at 15,000× g for 10 min to obtain the supernatant as the source of tyrosinase. The reaction mixture contained 20 mM phosphate buffer, pH 6.8, 1.25 mM l-dopa (Sigma-Aldrich, and the supernatant. After incubation at 37 °C for 30 min, dopachrome formation was monitored by measuring absorbance at a wavelength of 475 nm.

### 3.6. Instruments for in Vivo Measurements

The Chromameter CR-200 (Minolta, Osaka, Japan) is a colorimetric instrument containing a Xenon lamp as a light source. In this study, it was used to assess the skin-whitening index of the volunteers. The light reflected perpendicular to the skin is collected by photodetectors with colored filters for a tristimulus color analysis at 450, 560, and 600 nm, using the L*a*b* system, according to the CIE color systems [13]. We considered only the L* parameter. The L*-value gives the relative brightness, ranging from total black (L* = 0) to total white (L* = 100). The Chromameter necessitates a connection to a computer. After calibration on a white plate, the 8-mm probe is applied to the skin simply by the weight of the instrument. After the shutter button is pushed, the results can be immediately read on the monitor.

### 3.7. In Vivo Measurements

The study was approved by the Ethics Committee of the Chia Nan University of Pharmacy and Science. Thirty-five female volunteers, aged between 22 and 49 years with normal healthy skin, were included in the study. They were divided into four groups with treatments of placebo (five volunteers), 2% AA2G (five volunteers), 2% 8-OHDe (10 volunteers), and 4% 8-OHDe (15 volunteers). The investigation was performed in a double-blind and randomized fashion. All measurements were carried out in a climatized room under controlled ambient conditions (room temperature 25 ± 2 °C and relative humidity 45 ± 5%). An acclimatization period of at least 20 min was respected before the start of every measurement session. As an objective measurement of the color of skin, one type of tristimulus reflectance instrument, Chromameter CR-200, was used at each clinical visit. The initial skin-whitening index L*-value was taken from the cheek of each subject before application of the tested substances. The tested substances were then applied twice daily on the outer surface of the skin area of each volunteer. The application continued for 8 weeks, and the skin-whitening index was measured at the same skin area every week. During the measurement, the photoreceivers were placed perpendicularly on the skin with minimal pressure. Each spot was measured three times, and the average of the three measured values was calculated. All the experimental data were expressed as means ± S.D.

## 4. Conclusions

8-OHDe strongly inhibits tyrosinase with a unique suicide-inactivating mode. Until now, no tyrosinase suicide substrate has been confirmed to exhibit depigmenting activity either in cell cultures or in human skin. In the present study, 8-OHDe was demonstrated to exert potent inhibition in both melanogenesis and cellular tyrosinase activities in mouse B16 melanoma cells, where the IC_50_ values of the inhibitions by the compound are 10.54 and 6.17 μM, respectively. The depigmenting activity of 8-OHDe was more than 10-fold higher than a skin-whitening standard, kojic acid, in the cell-culture system. In addition, when a cream containing 4% 8-OHDe was applied on human skin, significant increases in the δL*-values were observed after three weeks. Moreover, the increase in the δL*-values after 8-week treatment with 4% 8-OHDe (from −0.57 to 1.94) is stronger than those of 2% 8-OHDe treatment (from 0.26 to 0.94) and 2% AA2G treatment (from 0.07 to 1.54). From the results of the study, it was concluded that 8-OHDe has depigmenting activities in both mouse melanoma cells and in human volunteers. Thus, the compound is a potential candidate for use in cosmetics as a skin-whitening ingredient.
